# Cardiopulmonary resuscitation in Brazilian medical television shows: a descriptive and quality assessment study

**DOI:** 10.62675/2965-2774.20250228

**Published:** 2025-05-13

**Authors:** Eduardo Messias Hirano Padrao, Fernando Onuchic, Monaliza de Almeida Castro, Ariadne Peres Silva Swarovsky, Augusto Barreto do Amaral, Felippe Lazar, Luciano César Pontes Azevedo, Fernando Godinho Zampieri, Caio de Assis Moura Tavares

**Affiliations:** 1 Harvard University Massachusetts General Hospital Department of Pulmonary and Critical Care Boston MA United States Department of Pulmonary and Critical Care, Massachusetts General Hospital, Harvard University - Boston, MA, United States.; 2 University of Connecticut Health Center Department of Internal Medicine Farmington CT United States Department of Internal Medicine, University of Connecticut Health Center - Farmington, CT, United States.; 3 Universidade Nove de Julho - Campus Vergueiro São Paulo SP Brazil Universidade Nove de Julho - Campus Vergueiro - São Paulo (SP), Brazil.; 4 Universidade do Vale do Rio dos Sinos São Leopoldo RS Brazil Universidade do Vale do Rio dos Sinos - São Leopoldo (RS), Brazil.; 5 Central Montana Medical Center Lewistown Montana United States Central Montana Medical Center - Lewistown, Montana, United States.; 6 Faculdade de Medicina, Universidade Hospital das Clínicas Instituto do Câncer São Paulo SP Brazil Instituto do Câncer, Hospital das Clínicas, Faculdade de Medicina, Universidade. de São Paulo - São Paulo (SP), Brazil.; 7 Hospital Israelita Albert Einstein Academic Research Organization São Paulo SP Brazil Academic Research Organization, Hospital Israelita Albert Einstein - São Paulo (SP), Brazil.; 8 University of Alberta Department of Critical Care Medicine Edmonton Canada Department of Critical Care Medicine, University of Alberta - Edmonton, Canada.; 9 Universidade de São Paulo Faculdade de Medicina Hospital das Clínicas São Paulo SP Brazil Geriatric Cardiology Unit, Instituto do Coração, Hospital das Clínicas, Faculdade de Medicina, Universidade de São Paulo - São Paulo (SP), Brazil.

**Keywords:** Heart arrest, Cardiopulmonary resuscitation, Prognosis, Quality of care, Television, Death, sudden, cardiac

## Abstract

**Objective:**

To assess the accuracy of Brazilian television depictions of cardiopulmonary arrest, their management, and outcomes and to compare the observed outcomes with prior data from observational studies.

**Methods:**

Investigators screened episodes, identified cardiac arrest scenes, collected relevant information, and assessed outcomes. Cardiac arrest scenes were then analyzed using the American Heart Association guidelines. The primary outcome was survival with favorable neurologic outcomes. Secondary outcomes were the return of spontaneous circulation and the number of Advanced Cardiovascular Life Support deviations in each event.

**Results:**

Fifty-nine cardiac arrests were included in the study. Death occurred in 55.9% of patients, and return of spontaneous circulation was obtained in 54.2%. Survival rate was 44.1%, and 42.4% of the patients had favorable neurologic outcomes. Adherence to Advanced Cardiovascular Life Support guidelines did not demonstrate a significant impact on survival with favorable neurological outcomes, as evidenced by comparable odds ratios (0.86 [95%CI 0.22 - 2.36] for 3 - 5 deviations and 0.69 [95%CI 0.07 - 5.93] for ≥ 6 deviations using 0 - 2 deviations as reference). Television shows depicted a significantly higher proportion of favorable outcomes than real-world Brazilian cohorts for out-of-hospital and in-hospital scenarios (50% *versus* 20.5%, p = 0.107; and 43.3% *versus* 17.4%, p < 0.0001, respectively).

**Conclusion:**

In Brazilian television shows, the portrayal of cardiopulmonary resuscitation is inaccurate and tends to overstate the likelihood of favorable outcomes following cardiac arrests.

## INTRODUCTION

Cardiac arrest is a devastating medical complication. It requires a fast and coordinated approach, named the chain of survival, to ensure the best patient outcome.^(1)^ Since cardiac arrest often occurs out-of-hospital, the approach requires bystanders, usually non-healthcare workers, to provide initial care to maximize patient outcomes.^(2)^ However, specific training of the lay population for essential life support is not mandatory in many countries, and less than 1% of the general population can perform it effectively.^(3)^ Less than 50% of witnessed out-of-hospital cardiac arrest cases receive bystander cardiopulmonary resuscitation (CPR), with even lower rates observed in non-white populations.^(4)^

One of the most common sources of health literacy among the general population is television (TV).^(5,6)^ Medical shows are typical on television or streaming platforms and are frequently watched by the population.^(7)^ Although medical shows are usually created with medical advice, the producers must adapt the clinical cases to make them more entertaining and appropriate to the script. Unfortunately, most of the TV shows do not follow the American Heart Association (AHA) or the European Resuscitation Council (ERC) recommendations.^(8–11)^

The depiction of outcomes in TV shows is often unrealistic,^(12,13)^ and the portrayal of palliative care is infrequent.^(10)^ This can impact the public's understanding of the seriousness, prognosis and appropriate management of cardiac arrest,^(14,15)^ as medical TV shows can serve as a source of health literacy. Cultural differences may also influence public perceptions, attitudes, and expectations regarding CPR. For example, physicians’ attitudes towards initiating CPR in cardiac arrest can be influenced by their cultural background,^(16)^ and end-of-life decisions can vary significantly across different cultures.^(17)^

To date, no research has been conducted on how Brazilian medical TV shows depict cardiac arrests in terms of the quality of resuscitation, adherence to Advanced Cardiovascular Life Support (ACLS) recommendations, or the prognosis following cardiac arrest. Therefore, this study aimed to assess the accuracy of Brazilian TV depictions of cardiopulmonary arrest, their management, and outcomes and to compare the observed outcomes with prior data from observational studies.

## METHODS

### Data collection

Two reviewers searched for Brazilian medical TV shows on Brazil's six leading streaming platforms: Globosat, Globoplay, Netflix, HBO Max, Disney+, and Amazon Prime. We did not include cable or free TV channels since we could not access their TV shows and soap operas. We used the keywords "doctor", "medic", "medical", "health", "med", "hospital" in both Portuguese and English in each of the platforms. The inclusion criteria were medical TV shows produced and conducted in Brazil without healthcare personnel education purposes. There were no language or year of production restrictions. Two reviewers watched all the TV shows and identified cardiac arrest cases based on explicit mentions or the initiation of chest compressions. A third reviewer resolved any discrepancies. For each occurrence of cardiac arrest, demographic data were collected, including age (reported or estimated), sex, initial setting, initial rhythm, etiology, provider of CPR, time and duration of the arrest, return of spontaneous circulation (ROSC), and outcome.

The identified cardiac arrest cases were then independently reviewed by two ACLS-certified providers, with involvement from a third author in case of disagreement, also ACLS-certified. The procedures evaluated included defibrillation, chest compression, airway management, drug administration, and pulse checking. A previously validated protocol,^(18,19)^ updated by Crowley et al.,^(20)^ was used to assess ACLS deviations. A comparison was made between the TV shows and the guidelines available during their production.^(1,21)^ We excluded cardiac arrests that were not shown, ACLS was not started, or the outcomes were unavailable. Neurologic outcomes were assessed using the Cerebral Performance Category scale (CPC), categorized as CPC 1 - 2 for favorable neurologic outcomes, CPC 3 - 4 for non-favorable neurologic outcomes, and CPC 5 for death.

### Study outcomes

The primary outcome was survival with favorable neurological outcomes after cardiac arrest, assessed at the end of the TV show episode. Favorable neurological outcomes were defined as CPC scores 1 and 2. We also examined ROSC and the number of ACLS deviations for each CPR occurrence. Detailed definitions of all criteria used for ACLS deviations are provided in table 1S (Supplementary Material).

### Statistical analysis

Summary statistics were used to describe the data, including means and standard deviations or medians and interquartile ranges (IQR) as appropriate. The number of events and proportions (%) were also reported. To evaluate the relationship between CPR quality and outcomes, the total number of deviations per CPR episode, according to ROSC and survival with favorable neurological outcome, were compared using the Wilcoxon-rank sum test for both outcomes. We also evaluated the association between grouped categories of ACLS deviations and survival with favorable neurological outcomes, using a logistic regression model, with ACLS deviations classified as low (0 - 2 deviations), medium (3 - 5 deviations), and high (≥ 6 deviations).^(20)^

An exploratory comparative analysis to examine the survival rates depicted in TV series and reported outcomes from Brazilian cohorts was conducted to compare the survival rates for out-of-hospital cardiac arrest and in-hospital cardiac arrest as shown on TV and in the real-world setting.^(22,23)^ The comparison was performed using a two-sample proportion z-test or the Fisher exact test, as appropriate. A two-sided alpha level of 5% was used as the statistical significance threshold for all statistical tests. No adjustments were performed for multiple comparisons. All statistical analyses were performed using R, version 4.1.3 or higher (R Foundation for Statistical Computing).

## RESULTS

### Television shows and population characteristics

**Figure 1:**
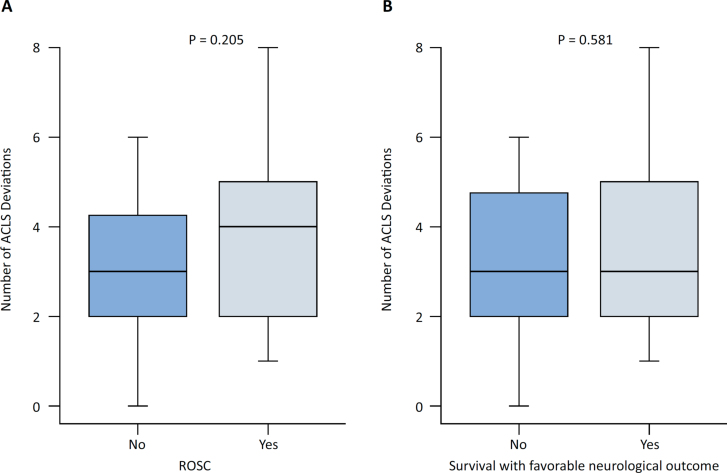
Box plots of Advanced Cardiovascular Life Support deviations per event according to the return of spontaneous circulation (A) and survival with favorable neurological outcome (B).

We obtained 79 medical TV shows after excluding 5 duplicates. We retrieved four TV shows on the leading streaming platforms. They were produced between 2014 and 2022. One was excluded because it is an educational TV show for medical students and not for the lay population. The three TV shows included were *Segredos Médicos*, *Sob Pressão* and *Unidade Básica*, accounting for 115 episodes. Out of the 65 identified cardiac arrests, a total of 59 cases were included in the study, after excluding cases in which CPR was not initiated (n = 2) or not depicted (n = 2), in which outcome information was unavailable (n = 1), and a cardiac arrest that had spontaneous resolution prior to CPR (n = 1). A flow diagram illustrating the selection process of TV shows and cardiac arrests is presented in figure 1S (Supplementary Material). The cardiac arrests, their episodes, and seasons are listed in table 2S (Supplementary Material).

### Cardiopulmonary resuscitation in Brazilian television shows

Among the 59 cardiac arrests analyzed, 23 cases (38.9%) were observed to be in a shockable rhythm, while 36 cases (61.1%) were in a non-shockable rhythm. The majority of the patients affected were young adults, with ages ranging from 21 to 50 years. Among the individuals included in the study, 62.7% were male. Most arrests were in-hospital cardiac arrest - accounting for 89.8% of the cases. In 40 cases (67.8%), physicians were identified as the leading providers initiating cardiac resuscitation. Discussions of goals of care were not performed in any of the cases. A comprehensive summary of the descriptive information about cardiac arrests is shown in table 1.

**Table 1:** Cardiopulmonary resuscitation in Brazilian TV shows

Characteristics		All patients N = 59	Survived n = 26	Death n = 33
Age* (year)				
	0 - 10	1 (1.7)	1 (3.8)	0 (0)
	11 - 20	6 (10.2)	5 (19.2)	1 (3)
	21 - 30	20 (33.9)	8 (30.8)	12 (36.4)
	31 - 40	14 (23.7)	5 (19.2)	9 (27.3)
	41 - 50	7 (11.9)	3 (11.5)	4 (12.1)
	51 - 60	6 (10.2)	2 (7.7)	4 (12.1)
	51 - 60	6 (10.2)	2 (7.7)	4 (12.1)
	61 - 70	4 (6.8)	2 (7.7)	2 (6.1)
	> 70	1 (1.7)	0 (0)	1 (3)
Sex				
	Male	37 (62.7)	15 (57.7)	22 (66.7)
	Female	20 (33.8)	9 (34.6)	11 (33.3)
	Other (non-binary)	2 (3.4)	2 (7.7)	0 (0)
Setting				
	In-hospital	53 (89.8)	23 (88.5)	30 (90.9)
	Out-of-hospital	6 (10.2)	3 (11.5)	3 (9.1)
Initial rhythm				
	Shockable	23 (38.9)	13 (50.0)	10 (30.3)
	Non-shockable	36 (61.1)	13 (50.0)	23 (69.7)
Initial provider				
	Physician	40 (67.8)	21 (80.8)	19 (57.6)
	Other	19 (32.2)	5 (19.2)	14 (42.4)
Other characteristics				
	Trauma	33 (55.9)	12 (46.2)	21 (63.6)
	Goals of care discussion	0 (0)	0 (0)	0 (0)

* Either provided or estimated age. Results expressed in n (%).

The most common cause identified for cardiac arrests was trauma, which accounted for 49.2% of the cases. Exogenous intoxication and hypoxemia were responsible for 8.5% of the cases each. Detailed information regarding the etiology of cardiac arrests is shown in table 2.

**Table 2:** Etiologies of cardiac arrests

Probable cause	
Trauma-related complications	29 (49.2)
Cardiogenic shock	1 (1.7)
Aortic dissection	1 (1.7)
Hyperkalemia	1 (1.7)
Septic shock	4 (6.8)
Exogenous intoxication	5 (8.5)
Hypoxemia	5 (8.5)
Electrical shock	2 (3.4)
Dengue fever	1 (1.7)
Pulmonary embolism	3 (5.1)
Non-traumatic hemorrhagic shock	2 (3.4)
Arrhythmogenic disorder	1 (1.7)
Neoplasia complication	1 (1.7)
Stroke	1 (1.7)
Diabetic ketoacidosis	1 (1.7)
Status epilepticus	1 (1.7)

Results expressed in n (%).

### Advanced Cardiovascular Life Support characteristics and deviations

Appropriate defibrillation was carried out in 22 cases of ventricular fibrillation or pulseless ventricular tachycardia, accounting for a rate of 95.7%. Conversely, defibrillation was appropriately withheld in 31 cases of pulseless electrical activity or asystole, representing a rate of 86.1%. Chest compressions were not administered in 13 cases, which accounts for approximately 22.0% of the total. Among the cases in which chest compressions were performed, the location was appropriate in 40 instances (67.8%), and the frequency was appropriate in 21 cases (35.6%). Regarding ventilation, 15 patients (25.4%) did not receive any form of ventilation. Among the remaining patients, 29 were intubated (49.2%), 14 underwent bag-valve-mask ventilation (23.7%), and one underwent cricothyrotomy. Only 33 patients (55.9%) received appropriate drug management during the cardiac arrest. Notably, no cardiac arrest cases involved appropriate pulse checking.

The median deviation from ACLS protocol was 5 (IQR 3 - 8) in patients with shockable rhythms and 2 (IQR 1 – 5) in patients with non-shockable rhythms. The total number of ACLS deviations per cardiac arrest is shown in figure 2S (Supplementary Material). Regarding delays in resuscitation, CPR was not immediately started in 36.2% of the cases, and 75.9% of the intubations delayed CPR. Defibrillation was incorrectly performed (wrong dose or frequency) in 47.8% of the cases. Drug omission or excess occurred in 46.2% of the cases, and the airway was not appropriately managed in 63.0% of the cases evaluated. Other findings regarding deviation from ACLS protocol are described in table 3. The deviations using the ACLS standardized evaluation are provided in table 4.

**Table 3:** General characteristics of Advanced Cardiovascular Life Support

Characteristics		Patients
Shock delivery		
	Appropriate shock delivered (VF or VT)	22/23 (95.7)
	Appropriate shock withheld (PEA or asystole)	31/36 (86.1)
	Chest compressions	
Chest compression delivered		46/59 (78.0)
	Appropriate frequency	21/59 (35.6)
	Appropriate location	40/59 (67.8)
Airway management		
	Appropriate CPR coordination*	4/59 (6.7)
	Intubation	29/59 (49.2)
	Bag-valve-mask	14/59 (23.7)
	Cricothyrotomy	1/59 (1.7)
	Mouth-to-mouth	0/59 (0.0)
	No ventilation	15/59 (25.4)
Drug administration		
	Epinephrine	31/59 (52.5)
	Amiodarone	3/59 (5.1)
	Other medication	0/59 (0.0)
	Appropriate drug†	33/59 (55.9)
Pulse checking		
	Appropriate‡	0/59 (0.0)
	Inappropriate	57/59 (96.7)
	Could not be evaluated	2/59 (3.3)

VF - ventricular fibrillation; VT - ventricular tachycardia; PEA - pulseless electrical activity; CPR - cardiopulmonary resuscitation.

* Considered 30:2 (compressions:ventilations) if patient not intubated or ventilations every 5 to 6 seconds if intubated;

† considered appropriate drug, sequence, and timing according to Advanced Cardiovascular Life Support guidelines (did not consider appropriate dose);

‡ considered appropriate if < 10 seconds duration, within the appropriate interval (2 minutes) and appropriate central pulse location (carotid or femoral arteries). Results expressed in n/total (%).

**Table 4:** Advanced Cardiovascular Life Support deviations standardized evaluation

ACLS deviations		Cardiac arrests
Delays in CPR (all cardiac arrests)		
	CPR not started within the exact minute of recognizing pulselessness	21/58 (36.2)
	CPR delayed > 10 seconds at pulse/rhythm check	5/21 (23.8)
	CPR delayed for endotracheal tube placement	22/26 (84.6)
Pulseless VT/VF algorithm deviations (only VT/VF)		
	Any indicated ACLS drug given before the second shock	5/23 (21.7)
	Incorrect dose of any indicated drug	2/23 (8.7)
	Incorrect sequence of any indicated drug	0/23 (0.0)
	Incorrect ACLS drug is given for pulseless VT/VF	2/23 (8.7)
	Drug omission or excess	2/23 (8.7)
	Delay > 1 min between rhythm recognition and shock delivery	2/23 (8.7)
	The shock delivered at incorrect voltage or no shock delivered at the appropriate interval	11/23 (47.8)
	Failure to resume CPR immediately after shock delivery	17/23 (73.9)
PEA/Asystole algorithm deviations (only PEA/Asystole)		
	Incorrect dose of any indicated drug	6/36 (16.7)
	Incorrect sequence of any indicated drug	1/36 (2.8)
	Incorrect ACLS drug given for PEA/asystole	1/36 (2.8)
	Delays in administering indicated drug	0/36 (0.0)
	Drug omission or excess	18/36 (50.0)
	Administered shock in a patient with PEA or asystolic rhythm	0/36 (0.0)
Other (all cardiac arrests)	
	Airway management not appropriately done	34/54 (63.0)

ACLS - Advanced Cardiovascular Life Support; CPR - cardiopulmonary resuscitation; VT - ventricular tachycardia; VF - ventricular fibrillation; PEA - pulseless electrical activity. Many events were not possible to be evaluated due to scene cuts, or cardiac arrest was not long enough to be thoroughly evaluated. Results expressed in n/total (%).

### Outcomes

Death occurred in 33 (55.9%) of the patients, while ROSC was obtained in 32 patients (54.2%). Among the 26 patients that survived, 25 (96.2%) had favorable neurological outcomes, and only one (3.8%) had a non-favorable neurological outcome. No significant differences were observed in median (IQR) of ACLS deviations per cardiac arrest episode in patients with ROSC and those who survived with favorable neurological outcomes (Figures 1A and 1B, respectively). There was no association between ACLS deviation categories and survival with favorable neurological outcomes. The crude OR for survival with favorable neurological outcome was 1.07 (95%CI 0.34 - 3.38) for 3-5 deviation categories and 1.95 (95%CI 0.41 - 9.84) for 6 or more deviation categories when using 0-2 deviation as reference (Table 3S - Supplementary Material).

The median (interquartile ranges) number of Advanced Cardiovascular Life Support deviations per event was analyzed based on the occurrence of return of spontaneous circulation (A) and survival with favorable neurological outcome (B). For cardiac arrests that did not result in the return of spontaneous circulation, the median number of Advanced Cardiovascular Life Support deviations was 3 (interquartile ranges 2 to 4), while for those that did achieve a return of spontaneous circulation, it was 4 (interquartile ranges 2 to 5; p = 0.205). Similarly, among patients who did not survive with a favorable neurological outcome, the median number of Advanced Cardiovascular Life Support deviations was 3 (interquartile ranges 2 to 5), whereas, for those who did survive favorably, it was also 3 (interquartile ranges 2 to 5; p = 0.581). Several Advanced Cardiovascular Life Support deviations are expressed as median, first, and third quartiles (boxes), and range (lines). ACLS - Advanced Cardiovascular Life Support; ROSC - return of spontaneous circulation.

### Exploratory relationship between survival rates in television series and observational cohorts

The survival rate observed in Brazilian TV shows for out-of-hospital cardiac arrest was numerically higher compared to the rate reported in Brazilian cohorts (50% *versus* 20.5%, respectively; p = 0.107). Similarly, for in-hospital cardiac arrest, the proportion of patients depicted as surviving in TV shows was significantly higher than the reported rate in reality (43.3% *versus* 17.4%, respectively; p < 0.0001). The results are shown in table 4S.

## DISCUSSION

In this study analyzing cardiac arrests in Brazilian TV shows produced from 2014 to 2022, we found that the median deviation from the ACLS protocol was five (IQR 3 - 8) in shockable rhythms and two (IQR 1 - 5) in non-shockable rhythms. Twenty-six patients (45.8%) who had cardiac arrest survived, and, among those, 25 (96.2%) had good neurologic outcomes. Death occurred in 33 patients (55.9%).

Our results are similar to the previous literature on medical TV shows, in which ROSC ranged from 25 to 69% and survival from 19 to 41%.^(7–9,11,12,22–25)^ Compared to real-world data, our TV show's rates of ROSC and survival are much higher. In-hospital cardiac arrest has survival to hospital discharge of less than 25%,^(26,27)^ while our data showed a short-term survival of 45.8%. In the out-of-hospital settings, although it occurred much less frequently in our study (six cases), we observed a 50% short-term survival rate, which is much higher when compared to the 10% reported survival in an observational study.^(26)^ Similarly, our study has a much higher number of patients with favorable neurologic outcomes. Only one patient had non-favorable neurologic outcomes, while the other 25 surviving patients (96.2%) had favorable neurologic outcomes, most with no sequelae. Compared to the outcomes observed in cohort studies, our results demonstrate a significantly higher occurrence of favorable neurological outcomes (CPC 1 to 2) among cardiac arrest survivors. These favorable outcomes are typically observed in only 20 to 40% of such cases.^(28–32)^

Goals of care discussion and palliative care were never involved in the TV shows examined, perhaps due to our study's high rate of ROSC and survival with favorable neurologic outcomes and demanding access to palliative care or cultural aspects.^(33,34)^ This result is similar to previous studies. To our knowledge, only one TV show study reported advanced care planning and goals of care discussions. In 91 episodes described, only 11 patients had advanced care planning.^(10)^

Regarding deviations from ACLS protocol, our study showed results similar to those of the medical literature. We obtained a median deviation of 5 for shockable rhythms and 2 for non-shockable rhythms. A retrospective study in a single center showed that 72.6% of 150 patients had 2 or more deviations from ACLS protocol, and 24% had 5 or more deviations.^(18)^ Another study found that the mean deviation for patients who survived cardiac arrest was 2.1 *versus* 3.3 for those who did not.^(19)^ Unlike previous literature, our study found no correlation between the mean deviation from ACLS and outcomes. Compared to other medical TV show studies, only one study assessed the quality of CPR. It revealed that only 35.3% of patients had the correct compression frequency, and 16.9% had the correct ventilation frequency.^(25)^ These findings suggest that the CPR quality in Brazilian TV shows may not be meaningful for favorable outcomes.

The etiologies of cardiac arrest in our study were primarily due to complications of trauma (55%), although most only occurred in hospital. The high amount of trauma population in the TV shows also may explain the high percentage of young adults. This is also unusual when compared to real-world data, in which the mean age of cardiac arrest is around 65 years old for both in-hospital and out-of-hospital cardiac arrest.^(23,26)^ The causes of cardiac arrest are also different. While cardiovascular and respiratory causes are the most common causes,^(26)^ our study shows that in Brazilian TV shows, the most common causes are related to trauma complications. This significant difference in the prevalence of depicted *versus* observed causes of cardiac arrest can be seen as a missed opportunity to offer relevant and informative content for the lay public.

Although usually for entertainment, medical TV shows impact the population's medical knowledge. The Brazilian TV shows analyzed in this study have overestimated survival and favorable neurologic outcomes and do not depict CPR appropriately, similar to other studies.^(8–12,32,35)^ This may affect the general understanding of families regarding the severity of a patient post-cardiac arrest and affect decision-making by patients, guardians, family, and even among recently trained nurses and nurse technicians.^(36)^ The portrayal of CPR in Brazilian TV shows could be considered a missed opportunity to effectively communicate essential CPR concepts to the general public.

Therefore, the current study's findings emphasize the need for stricter guidelines for presenting health information in the media and suggest that exposure to correct information about CPR may increase the likelihood of the public taking appropriate and timely action in emergencies. This study also points to the need for future research assessing the effectiveness of educational interventions in the media and how different program formats (talk shows, documentaries) influence the audience's retention of information about CPR and the development of guidelines for the production of health content in the media, based on best practices identified in the literature.

### Limitations

Our study has several limitations that need to be acknowledged. Firstly, despite conducting a comprehensive search, we only identified three Brazilian medical TV shows on streaming platforms, and we did not include movies, non-medical TV shows, or soap operas in our analysis. Expanding the search to include these sources could potentially increase the number of cardiac arrests identified, leading to a more comprehensive analysis and less bias. Secondly, we did not assess the impact of TV shows broadcasted on free and cable television sources, which typically have a larger audience reach. This aspect may be relevant to understanding how such shows influence the Brazilian population. Additionally, some of our analyses rely on extrapolation from international data, which may be unsuitable due to potential social and cultural differences between countries.

## CONCLUSION

Brazilian TV shows do not depict appropriate cardiopulmonary resuscitation and overestimate survival and favorable neurologic outcomes after cardiac arrests. This may affect the general understanding of the population regarding the severity of this particular type of patient post-cardiac arrest and affect decision-making.
