# Supplementary material for: Cardiopulmonary resuscitation in Brazilian medical television shows: a descriptive and quality assessment study
Source: Crit Care Sci. 2025 May 13;37:e20250228. doi: 10.62675/2965-2774.20250228 (PMC12266834; doi:10.62675/2965-2774.20250228)
Supplement: Supplementary file 1 [file 2965-2774-ccsci-37-e20250228-Suppl01.pdf]

Critical Care Science
https://doi.org/10.62675/2965-2774.20250228

SUPPLEMENTARY MATERIAL

Cardiopulmonary resuscitation in Brazilian medical television shows: a descriptive and quality assessment study

Eduardo Messias Hirano Padrao[1], Fernando Onuchic[2], Monaliza de Almeida Castro[3], Ariadne Peres Silva Swarovsky[4], Augusto Barreto do Amaral Neto[5], Felippe Lazar Neto[6], Luciano César Pontes Azevedo[7], Fernando Godinho Zampieri[8], Caio de Assis Moura Tavares[7,9]

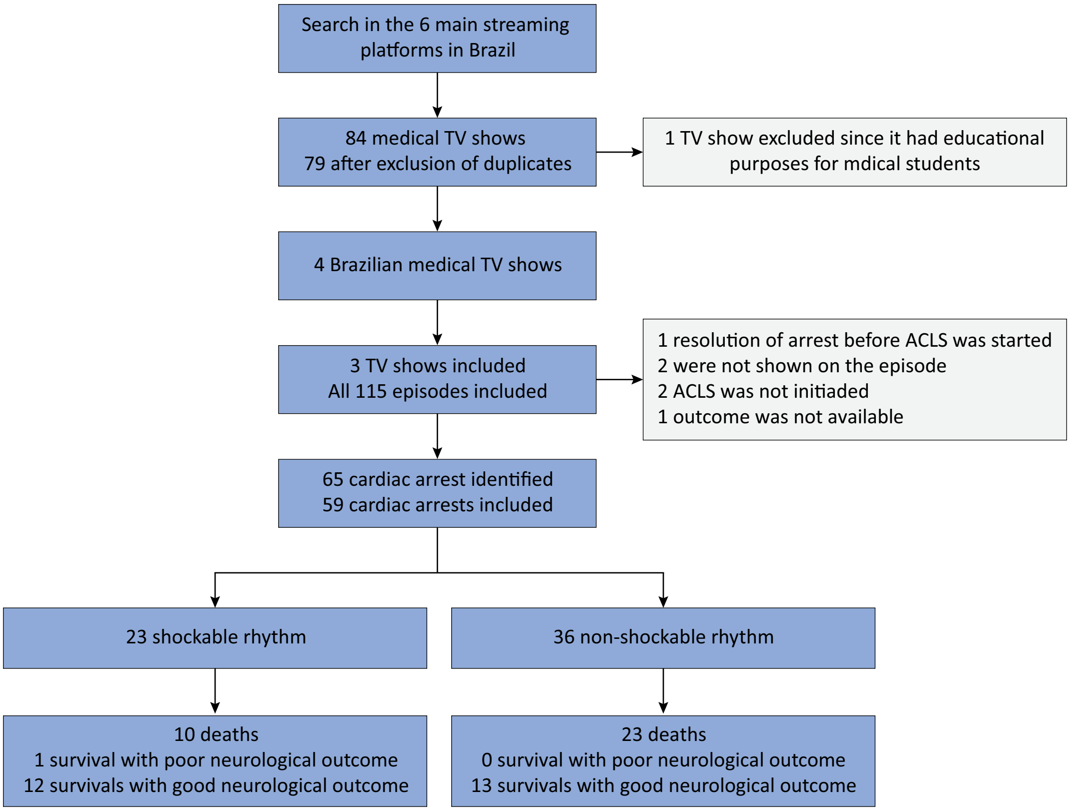

Figure 1S - Selection process of television shows and cardiac arrests.
ACLS - Advanced Cardiovascular Life Support.

This is an open access article under the CC BY license (https://creativecommons.org/licenses/by/4.0/).

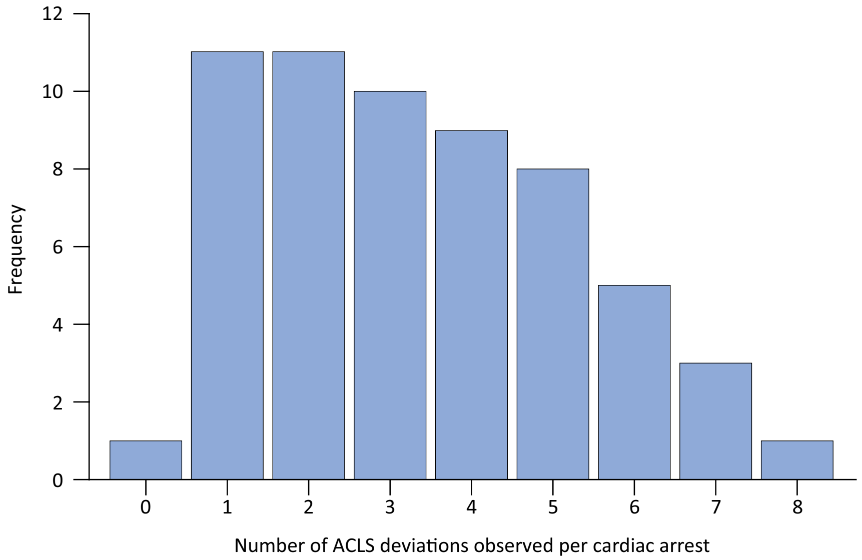

Figure 2S - Number of advanced cardiac life support deviations per cardiac arrest.
ACLS - Advanced Cardiovascular Life Support.

Table 1S - Form for evaluation of advanced cardiac life support deviations

Delays in CPR	– CPR not started within the same minute of recognizing pulselessness<br>– CPR delayed > 10 seconds at pulse/rhythm check<br>– CPR delayed for endotracheal tube placement
Pulseless VF/VT algorithm deviations	– Any indicated ACLS drug given before the second shock<br>– Incorrect dose of any indicated drug<br>– Incorrect sequence of any indicated drug<br>– Incorrect ACLS drug is given for pulseless VF/VT<br>– Drug omission or excess<br>– Delay > 1 minute between rhythm recognition and shock delivery<br>– The shock delivered at incorrect voltage or no shock delivered at the appropriate interval<br>– Failure to resume CPR immediately after shock delivery
PEA/asystole algorithm deviations	– Incorrect dose of any indicated drug<br>– Incorrect sequence of any indicated drug<br>– Incorrect ACLS drug given for PEA/ asystole<br>– Delays in administering indicated drug<br>– Drug omission or excess<br>– Administered shock in a patient with PEA or asystolic rhythm
Other	– Airway management indicated but not done

CPR - cardiopulmonary resuscitation; VF - ventricular fibrillation; VT - ventricular tachycardia; ACLS - Advanced Cardiovascular Life Support; PEA - pulseless electrical activity.

Table S2 - Total number of Advanced Cardiovascular Life Support deviations per cardiac arrest are

Number	Show	Season	Episode
1	*Sob Pressão*	1	1
2	*Sob Pressão*	1	2
3	*Sob Pressão*	1	3
4	*Sob Pressão*	1	6
5	*Sob Pressão*	1	6
6	*Sob Pressão*	1	6
7	*Sob Pressão*	1	9
8	*Sob Pressão*	2	1
9	*Sob Pressão*	2	2
10	*Sob Pressão*	2	2
11	*Sob Pressão*	2	2
12	*Sob Pressão*	2	3
13	*Sob Pressão*	2	4
14	*Sob Pressão*	2	4
15	*Sob Pressão*	2	5
16	*Sob Pressão*	2	6
17	*Sob Pressão*	2	8
18	*Sob Pressão*	2	9
19	*Sob Pressão*	2	10
20	*Sob Pressão*	3	2
21	*Sob Pressão*	3	3
22	*Sob Pressão*	3	5
23	*Sob Pressão*	3	4
24	*Sob Pressão*	3	6
25	*Sob Pressão*	3	6
26	*Sob Pressão*	3	8
27	*Sob Pressão*	3	8
28	*Sob Pressão*	3	11
29	*Sob Pressão*	3	13
30	*Sob Pressão*	3	13
31	*Sob Pressão*	3	14
32	*Sob Pressão*	COVID-19	1
33	*Sob Pressão*	COVID-19	1
34	*Sob Pressão*	COVID-19	2
35	*Sob Pressão*	4	1
36	*Sob Pressão*	4	1
37	*Sob Pressão*	4	2
38	*Sob Pressão*	4	4
39	*Sob Pressão*	4	5
40	*Sob Pressão*	4	5
41	*Sob Pressão*	4	6
42	*Sob Pressão*	4	8
43	*Sob Pressão*	4	8
44	*Sob Pressão*	4	9
45	*Sob Pressão*	5	4
46	*Sob Pressão*	5	6
47	*Sob Pressão*	5	7
48	*Sob Pressão*	5	7
49	*Sob Pressão*	5	8
50	*Sob Pressão*	5	9
51	*Sob Pressão*	5	10
52	*Sob Pressão*	5	11
53	*Unidade Básica*	1	4
54	*Segredos Médicos*	2	1
55	*Segredos Médicos*	2	5
56	*Segredos Médicos*	2	6
57	*Segredos Médicos*	2	7
58	*Segredos Médicos*	2	8
59	*Segredos Médicos*	2	9
60	*Segredos Médicos*	2	11
61	*Segredos Médicos*	2	11
62	*Segredos Médicos*	2	14
63	*Segredos Médicos*	2	15
64	*Segredos Médicos*	2	19
65	*Segredos Médicos*	2	20

Continue...

...continuation

Table 3S - Results of logistic regression analysis on survival with favorable neurological outcome according to protocol deviation categories

Deviation categories	Crude OR for survival with favorable neurological outcome (95%CI)
0 - 2	Ref
3 - 5	1.07 (0.34 - 3.38)
≥ 6	1.95 (0.41 - 9.84)

Association between survival with favorable neurological outcome according to the Advanced Cardiovascular Life Support deviation categories as proposed by Crowley CP et al.[20]
OR - odds ratio; 95%CI - 95% confidence interval.

Table 4S - Comparison of depicted survival rates on television shows and Brazilian cohorts

	TV shows	Brazilian cohorts	p value
Out-of-hospital cardiac arrest	3/6 (50)	239/1165 (20.5)	0.107
In-hospital cardiac arrest	23/53 (43.3)	400/2296 (17.4)	< 0.0001

Comparison between the survival rates depicted on TV shows and the reported survival rates in two Brazilian cohorts. The survival rates for out-of-hospital cardiac arrest were compared using a Fisher exact test, while a two-proportions Z-test was employed to compare in-hospital cardiac arrest. Results expressed in N/n (%).
